# 

**Published:** 1896-11

**Authors:** 


### BOOKS RECEIVED.

System of Diseases of the Eye. By American, British, Dutch, French, German and Spanish authors. Edited by William F. Morris, A. M., M. D., and Charles A. Oliver, A. M., M. D., of Philadelphia, Pa. Volume I. Embryology, Anatomy and Physiology of the Eye. Octavo, pp. xvi.—670. With twenty-three full-page plates and 362 text illustrations. Philadelphia: J. B. Lippincott Co. 1896.

A Text-Book of Materia Medica, Therapeutics and Pharmacology. By George Frank Butler, Ph. G., M. D., Professor of Materia Medica and Clinical Medicine in the College of Physicians and Surgeons, Chicago; Professor of Materia Medica and Therapeutics, Northwestern University, Woman's Medical School; attending Physician to Cook County Hospital, etc. Octavo, pp. 858. Price, $4 net. Philadelphia: W. B. Saunders, 925 Walnut street. 1896.

The Practice of Medicine. By Horatio C. Wood, A. M., M. D., Professor of Therapeutics and Clinical Professor of Nervous Diseases in the University of Pennsylvania, etc., and Reginald H. Fitz, A. M., M. D., Hersey Professor of the Theory and Practice of Physics in Harvard University, etc. Octavo, pp. x.—1088. Philadelphia: J. B. Lippincott Co. London: 10 Henrietta street, Covent Garden. 1896.

A Practical Treatise on Medical Diagnosis. For the use of Students and Practitioners. By John H. Musser, M. D., Assistant Professor of

Clinical Medicine, University of Pennsylvania, Philadelphia. New (second) edition, thoroughly revised. In one octavo volume of 925 pages, with 177 engravings and eleven full-page colored plates. Cloth, $5 ; leather, $6. Lea Brothers & Co., Publishers, Philadelphia and New York. 1896.

A Treatise on Obstetrics. For Students and Practitioners. By Edward P. Davis, A. M., M. D., Professor of Obstetrics and Diseases of Infancy in the Philadelphia Polyclinic ; Clinical Professor of Obstetrics in the Jefferson Medical College of Philadelphia. In one octavo volume of about 600 pages, with 217 engravings and 30 full-page plates in colors and monochrome. Cloth, $5 ; leather, $6. Lea Brothers & Co., Publishers, Philadelphia and New York. 1896.

A Text-Book of Diseases of the Nose and Throat. By Francke Huntington Bosworth, A. M., M. D. Illustrated with nearly 200 engravings and seven full-page chromo-lithographic plates. New York: William Wood & Company. 1896.

Transactions of the Medical Society of the State of Pennsylvania, at its Forty-sixth Annual Session, held at Harrisburg, 1896. Volume XXVII. Published by the Society. Wm. B. Atkinson, M. D., Secretary. Philadelphia : The Edwards & Docker Co., printers. 1896.

Transactions of the Medical Society of Virginia. Twenty-sixth Annual Session, held at Wytheville, Va., September 3, 4 and 5, 1895. Landon B. Edwards, M. D., Secretary. Richmond : J. W. Ferguson & Son, Printers. 1895.

A Text-Book for Training Schools for Nurses, including Physiology and Hygiene and the Principles and Practice of Nursing. By P. M. Wise, M. D., Medical Superintendent St. Lawrence Hospital ; Editor of *The State Hospitals Bulletin*, etc. With an introduction by Edward Cowles, M. D., Physician-in-Chief and Superintendent of the McLean Hospital, Boston, Mass. In two volumes. G. P. Putnam's Sons, 27 West Twenty-third street, New York. 1896.

Practical Notes on Urinary Analysis. By William B. Canfield, A. M., M. D., Lecturer on Clinical Medicine, University of Maryland, etc. Second revised edition. The Physician's Leisure Library. Detroit: George S. Davis. 1896.

